# Polymer-Based Scale Inhibition of Calcium Sulfate Using Phosphino-Polycarboxylic Acid: Experimental Evaluation, RSM Modeling, and Process Optimization

**DOI:** 10.3390/polym18101173

**Published:** 2026-05-10

**Authors:** Azizollah Khormali, Soroush Ahmadi

**Affiliations:** 1 Department of Chemistry, Faculty of Basic Sciences and Engineering, Gonbad Kavous University, Gonbad Kavous P.O. Box 4971799151, Iran; aziz.khormaly.put@gmail.com; 2 Department of Chemical Engineering, Faculty of Petroleum, Gas, and Petrochemical Engineering, Persian Gulf University, Bushehr P.O. Box 7516913817, Iran

**Keywords:** polymeric scale inhibitor, phosphino-polycarboxylic acid, calcium sulfate, scale inhibition efficiency, response surface methodology

## Abstract

Calcium sulfate scale formation is a major challenge in oilfield production systems. In this study, the performance of phosphino-polycarboxylic acid (PPCA) as a polymeric scale inhibitor was evaluated using static jar tests and modeled using Response Surface Methodology (RSM). The effects of temperature (50–100 °C), inhibitor concentration (10–50 ppm), and calcium ion concentration (1000–10,000 ppm) on inhibition efficiency (IE%) were investigated through 60 experimental runs. The results showed that IE% varied from 33.7% to 95.4%, depending on operating conditions. A quadratic RSM model demonstrated excellent predictive capability (R^2^ = 0.9977) with a low average error of 1.4%. Among the variables, inhibitor concentration had the strongest effect, followed by calcium ion concentration and temperature. Increasing PPCA concentration significantly improved inhibition efficiency, exceeding 90% at dosages above 35 ppm, while higher temperature and calcium concentration reduced performance. Response surface analysis indicated that IE% above 94% could be achieved at 43–50 ppm inhibitor and temperatures below 63 °C. Under harsh conditions (100 °C and 10,000 ppm Ca^2+^), 42–50 ppm PPCA maintained IE above 90%, with a predicted maximum of 90.81%. These results confirm the effectiveness of PPCA and provide a reliable basis for optimizing scale inhibition performance.

## 1. Introduction

Scale precipitation is a persistent and costly problem in oilfield operations and many industrial water systems. It occurs when dissolved ions in produced water or injected brines become supersaturated and form solid deposits [1,2,3,4]. In production systems, scale accumulates in tubing, valves, pumps, and surface facilities. This leads to pressure drops, equipment failure, and unplanned shutdowns [5,6,7]. The economic impact is significant due to lost production and high remediation costs [8,9,10,11]. Similar problems occur in desalination plants, cooling towers, and geothermal systems. Scale reduces heat transfer efficiency and increases energy consumption. It also shortens equipment lifetime and increases environmental burden due to frequent chemical cleaning [12,13,14]. In addition, chemical methods remain the most effective and widely used to control scale formation [15,16]. These include pH adjustment, precipitation control, and the use of scale inhibitors. Among these, scale inhibition is the most practical strategy [17,18,19]. It requires low chemical dosage and can be applied continuously or through squeeze treatments. Scale inhibitors delay nucleation, suppress crystal growth, and reduce deposition on surfaces. Compared to other methods, they offer long-term protection and flexibility. As a result, chemical inhibition has become the dominant technique in oilfield scale management [20,21,22,23].

Polymer-based scale inhibitors have attracted strong interest due to their high efficiency and tunable structure. These materials are macromolecules with repeating units that contain functional groups capable of interacting with ions and solid surfaces [24,25]. Functional groups such as carboxylate, phosphonate, and sulfonate provide active sites for binding with metal ions [26]. Among these materials, phosphino-polycarboxylic acid is widely used in oilfield applications [27,28,29]. PPCA contains both phosphino and carboxylic acid groups distributed along the polymer backbone. This dual functionality improves its ability to interact with calcium ions and crystal surfaces [30,31]. The inhibition mechanism of PPCA is closely related to its polymeric nature. The carboxylate and phosphinate groups can chelate calcium ions in solution. This reduces the concentration of free ions available for precipitation [32,33]. Another mechanism is adsorption on crystal surfaces. The polymer chains can attach to active growth sites on calcium sulfate crystals. This blocks further attachment of ions and slows down crystal growth. In addition, the polymer can modify crystal morphology [34,35,36]. Adsorbed polymer chains disrupt the regular arrangement of ions in the crystal lattice. This leads to distorted and less compact crystals that are easier to disperse. The efficiency of PPCA is therefore linked to its chain length, charge density, and distribution of functional groups [26,37,38,39].

The performance of polymeric scale inhibitors is influenced by several operational parameters [40,41]. Temperature is one of the most critical factors. An increase in temperature affects both polymer stability and scaling kinetics. Higher temperatures can accelerate crystal growth and reduce the effectiveness of adsorption [42,43]. They can also influence polymer conformation in solution. Reagent concentration is another key variable. At low concentrations, the polymer may not provide sufficient coverage of crystal surfaces. As concentration increases, inhibition efficiency improves due to increased availability of active sites [32,44]. Moreover, brine chemistry strongly affects scale inhibitor performance. Calcium ion concentration is particularly important in calcium sulfate scaling systems [40,45]. Higher calcium levels increase supersaturation and promote rapid nucleation. This requires more inhibitor to achieve effective control [46,47]. Therefore, the combined effect of temperature, inhibitor concentration, and brine composition must be carefully evaluated to optimize performance.

A detailed investigation by Farooqui and Sorbie [32] demonstrated that PPCA is highly effective in precipitation squeeze treatments. Also, it forms a sparingly soluble complex with calcium ions. This complex, often referred to as PPCA-Ca, plays a key role in controlling inhibitor release and long-term scale prevention. Their work revealed that the solubility behavior of this complex is not constant. Instead, it depends on exposure to fresh brine, indicating a dynamic dissolution mechanism rather than a simple equilibrium process. They proposed a “stripping” model. In this model, different molecular weight fractions of PPCA dissolve at different rates, significantly influencing the inhibitor return profile and its efficiency. This highlights a key polymer concept. The performance of PPCA is not governed solely by its chemical composition, but also by its chain length distribution and polydispersity. In addition to oilfield applications, polymeric inhibitors such as PPCA have also been studied in crystallization systems. A recent study by Lages et al. [48] on calcium carbonate precipitation demonstrated that polymeric inhibitors significantly alter nucleation and crystal growth processes. It was shown that inhibitors reduce calcium ion consumption, modify particle size distribution, and change crystal morphology. Specifically, PPCA was observed to reduce particle growth and produce smaller, less compact crystals. This behavior is attributed to adsorption of polymer chains onto active crystal growth sites, which disrupts lattice formation and prevents further ion attachment. In the study by Khormali et al. [49], the inhibition performance of PPCA is be explained by several simultaneous processes. First, the carboxylate and phosphino functional groups in the polymer backbone interact strongly with calcium ions through complexation. Second, the polymer chains adsorb onto crystal surfaces, blocking active growth sites. Third, steric hindrance created by the polymer chains prevents further approach of ions and clusters. These combined effects lead to delayed nucleation, reduced crystal growth rate, and formation of distorted or non-adherent scale deposits.

Beyond experimental evaluation, statistical and modeling approaches have become increasingly important in understanding and optimizing scale inhibition systems [50,51]. Among these, RSM has been widely applied due to its ability to model complex, nonlinear interactions between multiple variables. Several studies have demonstrated the effectiveness of RSM in analyzing the performance of corrosion and scale inhibitors under varying operational conditions. For example, Yamin et al. [52] applied RSM to evaluate the inhibition efficiency of an organic corrosion inhibitor under different temperatures and concentrations. Their results showed that RSM could successfully identify the optimal conditions. It also accurately predicted inhibition performance, with strong agreement between experimental and modeled data. The study also confirmed that inhibitor concentration and temperature are the most influential parameters. This finding is consistent with typical polymer adsorption behavior. Similarly, recent work [53] on polymeric scale inhibitors, such as maleic acid-acrylamide copolymers, has demonstrated the usefulness of RSM in analyzing scale inhibition efficiency. In this study, the effects of temperature, pH, and inhibitor concentration on calcium carbonate scale inhibition were systematically evaluated. The results showed that both linear and quadratic effects of the variables are significant. In addition, strong interaction effects exist between operating parameters. In the context of oilfield scale problems, RSM has also been applied to model precipitation and inhibition phenomena under dynamic conditions. For instance, Khormali and Ahmadi [54] on barium sulfate scaling using tube blocking tests have used RSM and ANOVA to develop predictive correlations for pressure drop and scale formation. These studies confirmed that statistical models can effectively capture complex relationships. These relationships exist between operational parameters such as flow rate, time, and ion concentration.

Despite the significant progress in both experimental and modeling studies, there are still gaps in the literature. Most previous works have focused either on the chemical performance of PPCA or on statistical modeling of scale inhibition using other types of inhibitors. Limited studies have combined detailed experimental evaluation of PPCA performance with advanced statistical modeling techniques such as RSM and ANOVA. In addition, the development of predictive correlations that explicitly incorporate key operational parameters such as temperature, inhibitor concentration, and calcium ion concentration remains limited. Therefore, the present study aims to address these gaps by integrating experimental investigation of PPCA performance in calcium sulfate systems with comprehensive statistical modeling. By applying RSM and ANOVA, a robust quadratic model is developed to describe the inhibition efficiency as a function of key variables. Furthermore, the study proposes a new empirical correlation for predicting inhibition efficiency and identifies optimal operating conditions for maximizing performance. This combined approach provides both mechanistic understanding and practical tools for the effective application of polymeric scale inhibitors in industrial systems.

## 2. Materials and Methodology

### 2.1. Materials

**Figure 1:**
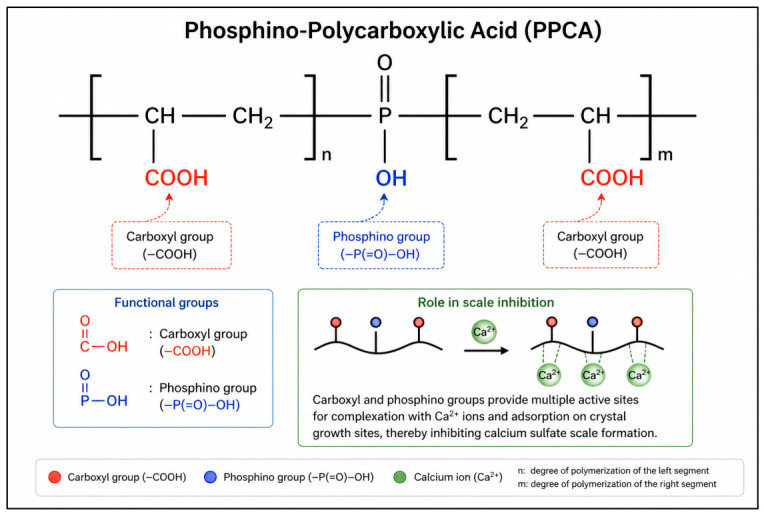
Schematic representation of the molecular structure of PPCA, illustrating the distribution of phosphino and carboxyl functional groups responsible for calcium ion complexation and scale inhibition.

The scale inhibitor used in this study was phosphino-polycarboxylic acid (PPCA). The polymer was supplied as an aqueous solution containing 42.2% (*w*/*w*) active inhibitor under the commercial name Bellasol by Italmatch Chemicals S.p.A., Naples, Italy. The reported purity of the active polymer content was higher than 95 wt%, with the remaining fraction consisting of water and trace stabilizers. The average molecular weight of PPCA was in the low-to-medium range (typically 1000–5000 g/mol), ensuring good solubility and effective transport in aqueous systems. Moreover, to provide a clearer understanding of the chemical characteristics of the inhibitor, a schematic representation of the PPCA molecular structure, including its and carboxyl functional groups, is presented in Figure 1. These functional groups play a key role in the inhibition mechanism through complexation with calcium ions and adsorption onto crystal growth sites [55].

Synthetic brines were prepared to simulate formation water and injection water conditions with controlled and reproducible compositions. Analytical-grade CaCl_2_·2H_2_O and Na_2_SO_4_ were used along with NaCl, KCl, and MgCl_2_ salts to incorporate major ions typically present in oilfield brines. Deionized water was used for all preparations. Three formation waters were prepared with identical ionic compositions except for calcium ion concentration (1000, 6000, and 10,000 ppm), while a single injection water rich in sulfate ions (2043 ppm) was used. The detailed ionic compositions of all brines are presented in Table 1.

All experiments were conducted by mixing formation water and injection water at a fixed volume ratio of 50:50, consistent with previous studies [56]. It should be noted that the calcium concentrations reported for the formation waters correspond to pre-mixing conditions; therefore, the effective calcium concentration in the mixed solution is approximately half of these values. This approach reflects realistic oil reservoir conditions, where formation water and injected water mix in situ, rather than controlled reactor or refinery environments.

The prepared brines contain high total dissolved solids (TDS) values (122,000–131,000 ppm for formation waters), reflecting high ionic strength conditions relevant to oilfield systems. Major ions such as Na^+^, K^+^, Mg^2+^, and Cl^−^ were included to account for ionic strength and charge shielding effects on polymer performance. However, ions such as Ba^2+^, Sr^2+^, CO_3_^2−^, and HCO_3_^−^ were deliberately excluded to prevent competing precipitation reactions and unintended consumption of calcium or sulfate ions. This controlled system allows for a more accurate evaluation of PPCA performance specifically for calcium sulfate scale inhibition.

The ionic composition of the prepared brines was controlled to ensure reproducibility. The pH of all solutions was adjusted to neutral conditions unless otherwise specified. All reagents were stored at ambient temperature and used within a short time to avoid any changes in composition.

### 2.2. Static Jar Test Method

The scale inhibition performance of PPCA was evaluated using a standard static jar test method. This method is widely used to assess the ability of inhibitors to prevent calcium sulfate precipitation under controlled laboratory conditions [57].

In a typical experiment, equal volumes of formation water (containing calcium ions) and injection water (containing sulfate ions) were mixed in glass bottles. Prior to mixing, the desired concentration of PPCA inhibitor (10–50 ppm) was added to the formation water. The mixture was then transferred into sealed glass bottles and placed in a thermostatically controlled oven. The experiments were conducted over a temperature range of 50 to 100 °C to simulate reservoir conditions. The samples were maintained at the target temperature for a fixed period, typically 24 h, to allow sufficient time for precipitation and equilibration. After incubation, the samples were cooled to room temperature. The solutions were then filtered to remove any precipitated solids. The inhibition efficiency (IE%) was calculated based on the concentration of calcium ions remaining in solution before and after the test. The following equation was used:

(1) $$IE=\frac{{\left[{Ca}^{2+}\right]}_{3}-{\left[{Ca}^{2+}\right]}_{2}}{{\left[{Ca}^{2+}\right]}_{1}-{\left[{Ca}^{2+}\right]}_{2}}\times 100$$

where ${\left[{Ca}^{2+}\right]}_{1}$ is the initial calcium ion concentration (ppm), ${\left[{Ca}^{2+}\right]}_{2}$ is the calcium ion concentration after precipitation without inhibitor, ${\left[{Ca}^{2+}\right]}_{3}$ is the calcium ion concentration after precipitation in the presence of inhibitor.

Calcium ion concentration was measured using a titrimetric method based on ethylenediaminetetraacetic acid (EDTA), which is widely accepted for its accuracy and simplicity [58]. A measured volume of the sample solution was taken after filtration. A buffer solution (typically ammonia buffer, pH = 10) was added to maintain the required pH for the titration. A small amount of Eriochrome Black T indicator was then introduced, resulting in a red color due to the formation of a calcium-indicator complex. Also, the solution was titrated with a standard EDTA solution of known concentration. As the titration proceeds, EDTA complexes free calcium ions, and at the endpoint, it displaces the indicator, producing a distinct color change from red to blue [57]. In addition, the endpoint was determined visually under controlled conditions by a single trained operator to ensure consistency. Standard calcium solutions and blank titrations were periodically conducted to verify the reliability of endpoint detection. Moreover, all measurements were performed in triplicate, and the average values were reported. The low deviation among replicate measurements confirms the precision of the method and indicates that potential interferences from polymer presence or high calcium concentration were negligible.

### 2.3. Response Surface Methodology

RSM is a powerful statistical tool that combines experimental design, regression modeling, and optimization techniques [59]. It enables the investigation of multiple variables simultaneously while minimizing the number of experimental runs. RSM was employed in this study to systematically design the experiments, evaluate the effects of key operational parameters, and develop a predictive model for the inhibition efficiency of phosphino-polycarboxylic acid in calcium sulfate scaling systems. In this study, the experimental design and statistical analysis were performed using Design-Expert^®^ software (Version 13.0.5.0).

In this work, three independent variables were selected based on their known influence on scale formation and polymer performance: temperature (A), inhibitor concentration (B), and calcium ion concentration (C). The selected ranges for these variables were chosen to represent realistic oilfield conditions. Temperature was varied from 50 to 100 °C in 3 levels, inhibitor concentration from 10 to 50 ppm in 5 levels, and calcium ion concentration from 1000 to 10,000 ppm in 4 levels (as shown in Table 2). It should be noted that the values −1 and +1 represent the coded levels of the independent variables used in the RSM design, where −1 represents the minimum level and +1 represents the maximum level of each factor. For example, for temperature three levels were considered and coded as −1, 0, and +1, corresponding to 50, 75, and 100 °C and for inhibitor concentration, five levels were used and coded as −1, −0.5, 0, +0.5, and +1, representing the selected range of PPCA concentration (10, 20, 30, 40, and 50 ppm).

In this study, a total of 60 experimental runs were conducted to ensure adequate coverage of the design space and to capture the nonlinear behavior of the system. Each experimental run corresponds to a unique combination of temperature, inhibitor concentration, and calcium ion concentration, as presented in the design matrix (Table A1 in Appendix A). The response variable measured was the inhibition efficiency (IE%) of calcium sulfate precipitation under static conditions. The experiments were performed according to the procedure described in the static jar test method. All experimental runs were conducted in triplicate to ensure reproducibility. The reported inhibition efficiency values represent the average of three independent measurements for each run. Furthermore, error and variance analyses were performed, showing that the deviations among replicate results were small for all 60 runs, confirming the high precision and reliability of the experimental data.

Moreover, the experimental values of PPCA scale inhibition performance have been examined using RSM through the following relationship [60]:

(2) $$Y=\beta_{0}+\sum \beta_{i}X_{i}+\sum \beta_{ii}X_{i}^{2}+\sum \sum \beta_{ii}X_{i}X_{j}\;\;\;\;\;(i\;and\;j\;=\;1,\;2,\;3,\text{…}\;K)$$

where *Y* shows the response (IE of PPCA), *X_i_* and *X_j_* depict the ith and jth input parameters, *β*_0_ is the intercept term, *β_i_* and *β_ii_* are linear and quadratic coefficients, and *β_ij_* depicts interaction coefficients between two different input parameters.

## 3. Results and Discussion

In this section, the influence of temperature (A), PPCA inhibitor concentration (B), and calcium ion concentration (C) on the inhibition efficiency (IE%) of calcium sulfate scale formation was systematically analyzed. The experimental results PPCA inhibition efficiency in 60 runs are shown Table 3. It should be noted that the temperature, PPCA concentration and calcium ion content of each run is demonstrated in Table A1. As depicted in Table 3, the inhibition efficiencies ranged from approximately 33.7% to 95.4%, depending on the operating conditions. Furthermore, a quadratic model based on RSM was developed to describe the system behavior and determine optimal operating conditions. The statistical analysis is supported by ANOVA results, model fit statistics, prediction errors, and diagnostic Figures.

### 3.1. ANOVA

ANalysis Of VAriance (ANOVA) is a statistical method utilized to determine the significance of developed models and laboratory parameters based on *p*-values and F-values [54]. The F-value reflects the strength of the model, while the *p*-value determines the statistical significance of each term. Furthermore, the model is considered highly significant if the *p*-value is less than 0.05 and the F-value is high. Table 4 represents the main results of ANOVA for the inhibition efficiency (IE%) of calcium sulfate scale formation using PPCA.

The ANOVA results presented in Table 4 clearly demonstrate that the quadratic model is highly significant. The model *p*-value is far below 0.05 (*p* < 0.0001), indicating that the relationship between the selected variables and inhibition efficiency is statistically meaningful and not due to random noise [61]. In addition, the very large F-value (4674.68) confirms the robustness of the model, with only about a 0.01% probability that such a value could occur due to random variation.

Examination of individual terms in Table 4 shows that all three main factors, temperature (A), inhibitor concentration (B), and calcium concentration (C), significantly influence the inhibition efficiency. Furthermore, the quadratic terms of temperature (A^2^) and inhibitor concentration (B^2^) are also significant, confirming the nonlinear nature of the system. This curvature is consistent with polymer adsorption behavior and the complex kinetics of calcium sulfate crystallization. A comparison of F-values in Table 4 indicates that inhibitor concentration (B) is the most influential parameter, followed by its quadratic term (B^2^), calcium concentration (C), temperature (A), and finally the quadratic temperature term (A^2^). Therefore, the relative importance of parameters can be ranked as: B > B^2^ > C > A > A^2^.

This result highlights the dominant role of PPCA concentration in controlling inhibition efficiency through adsorption and interaction with crystal growth sites.

### 3.2. Model Adequacy and Fit Quality

The adequacy and predictive capability of the developed model were evaluated using statistical parameters summarized in Table 5. These include the coefficient of determination (R^2^), adjusted R^2^ (Adj-R^2^), predicted R^2^ (Pred-R^2^), coefficient of variation (C.V), and adequate precision.

The R^2^ value of 0.9977 indicates that 99.77% of the variation in inhibition efficiency is explained by the model. This confirms an excellent agreement between experimental data and model predictions [62]. Additionally, the Adj-R^2^ (0.9975) and Pred-R^2^ (0.9971) values are in very close agreement, with a difference of less than 0.2%. This demonstrates that the model is highly reliable and has strong predictive capability without overfitting. The adequate precision value reported in Table 5 is 194.1393, which is significantly higher than the minimum acceptable value of 4. This indicates a very high signal-to-noise ratio and confirms that the model can effectively navigate the design space. Moreover, the coefficient of variation (C.V) is 2.37%, which is relatively low. This low variability reflects the high precision and reproducibility of the experimental data, further validating the developed model [63]. Based on the RSM, the following reduced quadratic model (in terms of Coded Factors) for Inhibition Efficiency was developed:

(3) $${IE}^{2.2}=16914.9\;-\;619.145A\;+\;9346.43B\;-\;838.855C\;+\;410.63A^{2}-\;4721B^{2}$$

### 3.3. Diagnostic Evaluation of the Model

**Figure 2:**
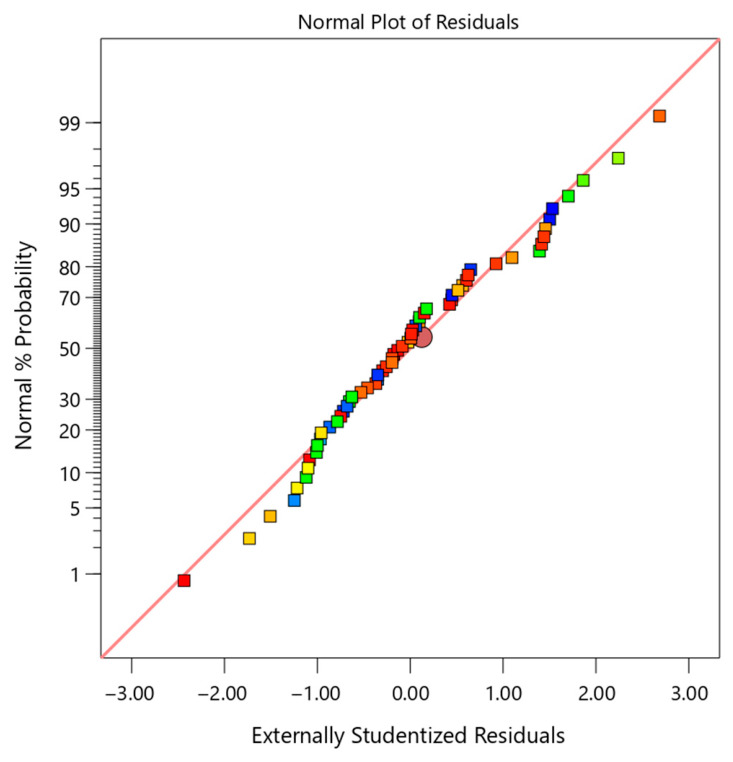
Normal probability plot of studentized residuals for the RSM model, showing linear alignment and confirming normality assumption.

**Figure 3:**
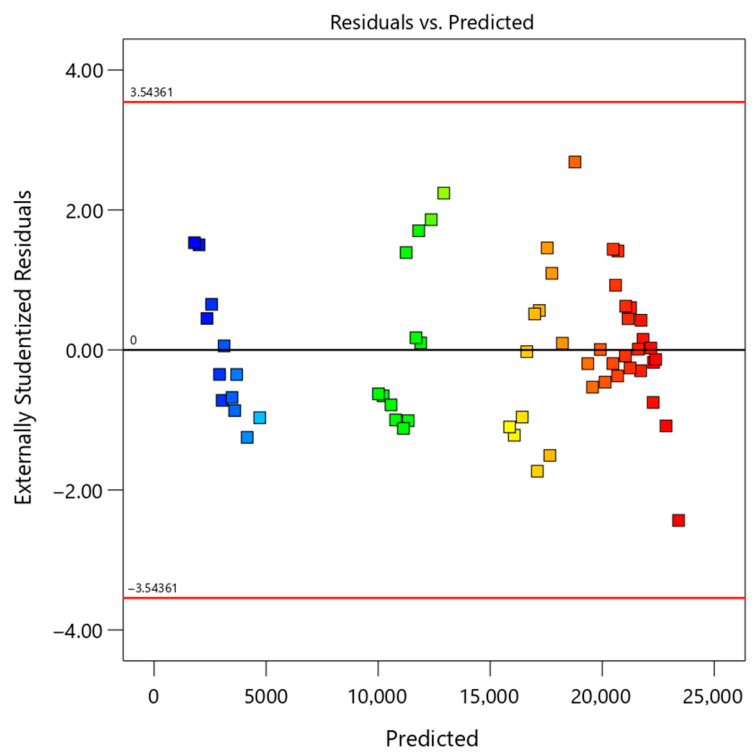
Residuals versus predicted values for the RSM model, indicating random scatter and validating model adequacy and variance consistency.

**Figure 4:**
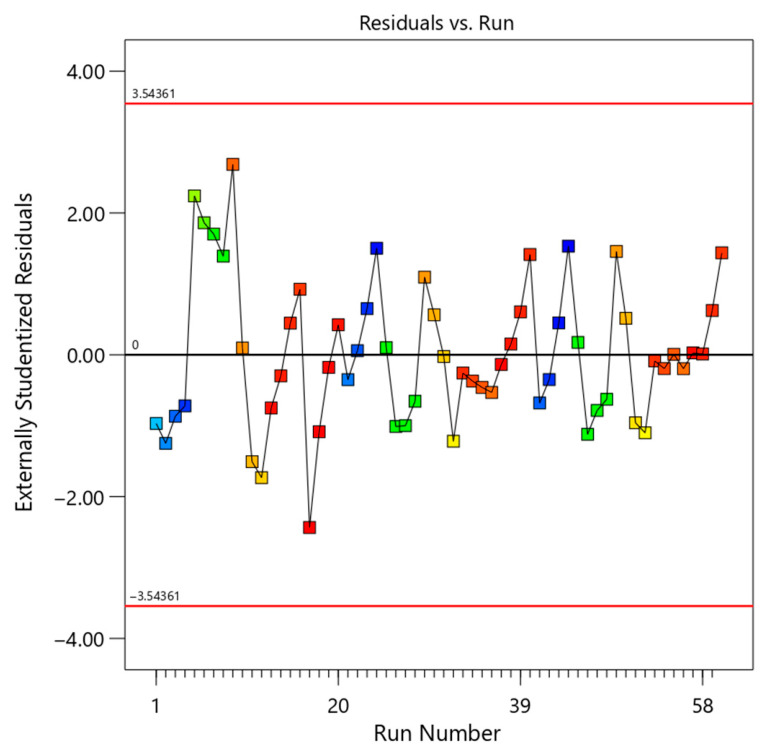
Residuals versus run order for the RSM model, confirming independence of observations and absence of systematic experimental bias.

**Figure 5:**
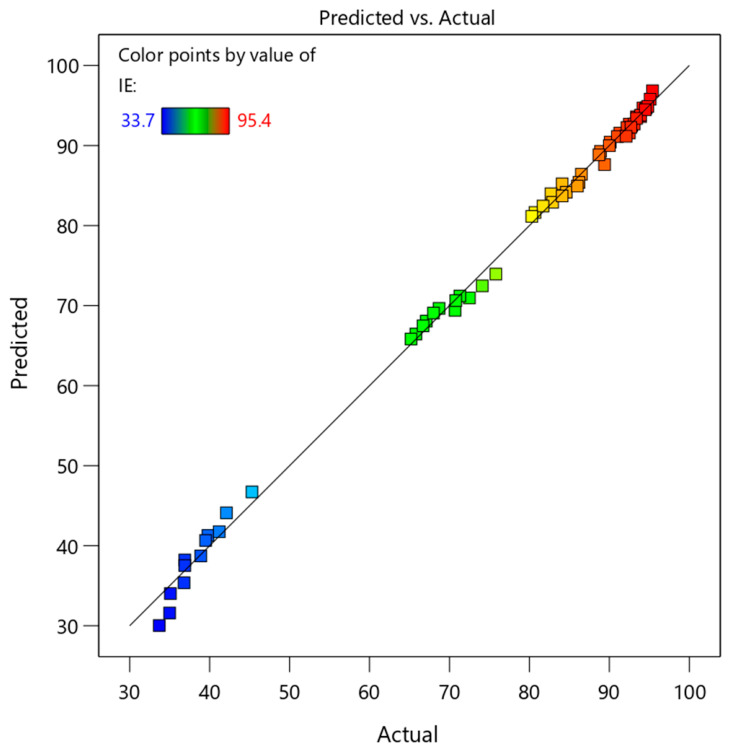
Predicted versus actual values for the RSM model, showing strong agreement and high accuracy of model predictions.

To further assess the validity of the model, several diagnostic plots were analyzed, as presented in Figure 2, Figure 3, Figure 4 and Figure 5. These figures provide a comprehensive evaluation of residual distribution, model assumptions, and agreement between predicted and experimental values.

Figure 2 shows the normal probability plot of studentized residuals. The data points closely follow a straight diagonal line, indicating that the residuals are normally distributed. This confirms that the fundamental assumption of normality required for ANOVA is satisfied. In the normal probability plot, residuals are colored based on inhibition efficiency, where blue indicates low values and red indicates high values; the random color distribution along the straight line confirms normality and validity of the RSM model assumptions.

Figure 3 presents the residuals versus predicted values plot. The residuals are randomly scattered around zero without any discernible pattern, indicating constant variance and absence of systematic error. This behavior confirms that the model adequately describes the experimental data across the entire range of predicted values. In the residuals versus run order plot, colors represent inhibition efficiency (blue: low, red: high); the random distribution of colored points around zero without clustering indicates independence of errors and stability of the RSM model.

Figure 4 illustrates the residuals versus run order. The random distribution of points without any trend demonstrates that the residuals are independent and that no external or time-dependent factors influenced the experimental results. This confirms the consistency and reliability of the experimental procedure.

Figure 5 shows the correlation between predicted and experimental inhibition efficiency values. The data points are closely aligned along the diagonal line, indicating excellent agreement between measured and predicted results. It should be noted that the color gradient represents inhibition efficiency (IE%), where blue indicates low efficiency (~33–47%), green represents moderate efficiency (65–75%), yellow to orange indicates higher efficiency (~80–85%), and red corresponds to the highest efficiency (~90–95%). Quantitatively, the prediction errors summarized in Table 6 show an average deviation of approximately 1.4%, which confirms the high accuracy of the developed model.

Based on the comprehensive analysis of Table 4 (ANOVA), Table 5 (fit statistics), Table 6 (prediction errors), and Figure 2, Figure 3, Figure 4 and Figure 5 (diagnostic plots), it can be concluded that the developed quadratic model is highly robust and reliable.

### 3.4. Effect of Operating Parameters on Inhibition Efficiency

The one-factor plots provide clear insight into the individual influence of temperature, phosphino-polycarboxylic acid concentration, and calcium ion concentration on inhibition efficiency under static conditions. In these diagrams, one parameter is varied while the other two are kept constant at their central values. This allows a direct interpretation of each variable in relation to polymer behavior and formation mechanisms [64].

#### 3.4.1. Effect of Temperature

**Figure 6:**
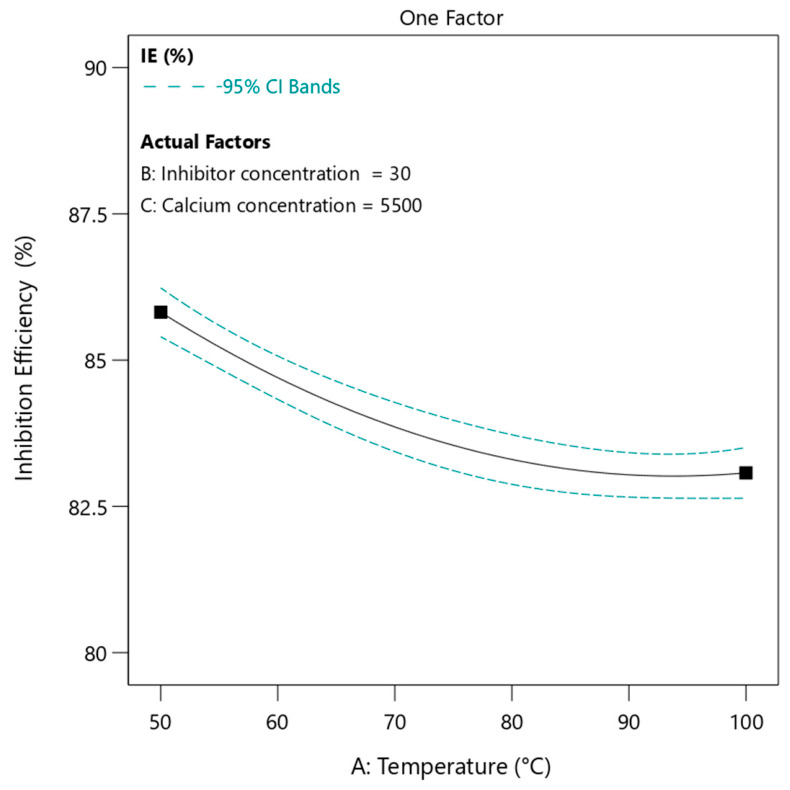
The effect of temperature on inhibition efficiency of PPCA at 30 ppm of inhibitor and 5500 ppm of calcium ion content under static condition.

The variation of inhibition efficiency with temperature in Figure 6 shows a decreasing trend as temperature increases from 50 to 100 °C. Quantitatively, the inhibition efficiency decreases from approximately 87% at 50 °C to about 83% at 100 °C. This change is not linear. The reduction in efficiency is more pronounced at lower temperatures, while at higher temperatures the slope becomes much smaller. For instance, there is almost no significant change in inhibition efficiency between 90 and 100 °C. In this case, the inhibitor concentration and calcium ion concentration were kept constant at 30 ppm and 5500 ppm, respectively.

This nonlinear behavior can be explained by the interplay between scale formation kinetics and polymer adsorption characteristics. At lower temperatures, calcium sulfate nucleation and growth are relatively slow, so small increases in temperature significantly enhance ion mobility and supersaturation-driven processes [37]. This results in a noticeable reduction in inhibition efficiency because the rate of scale formation begins to compete more effectively with the inhibition mechanisms of PPCA. At higher temperatures, however, the system approaches a regime where the polymer adsorption layer has already reached a reduced effectiveness due to thermal effects [15]. In this region, further increases in temperature do not significantly alter the already weakened adsorption equilibrium. From a polymer science perspective, the conformation of phosphino-polycarboxylic acid chains becomes less favorable for strong surface attachment at elevated temperatures [49]. Thermal motion weakens hydrogen bonding and electrostatic interactions between functional groups and calcium ions. Additionally, at higher temperatures, the adsorption-desorption equilibrium tends to stabilize. The polymer layer on the crystal surface becomes relatively thin but stable, leading to a plateau-like behavior in inhibition efficiency. This explains why the efficiency reduction is steep at lower temperatures but minimal at higher temperatures.

#### 3.4.2. Effect of Inhibitor Concentration

**Figure 7:**
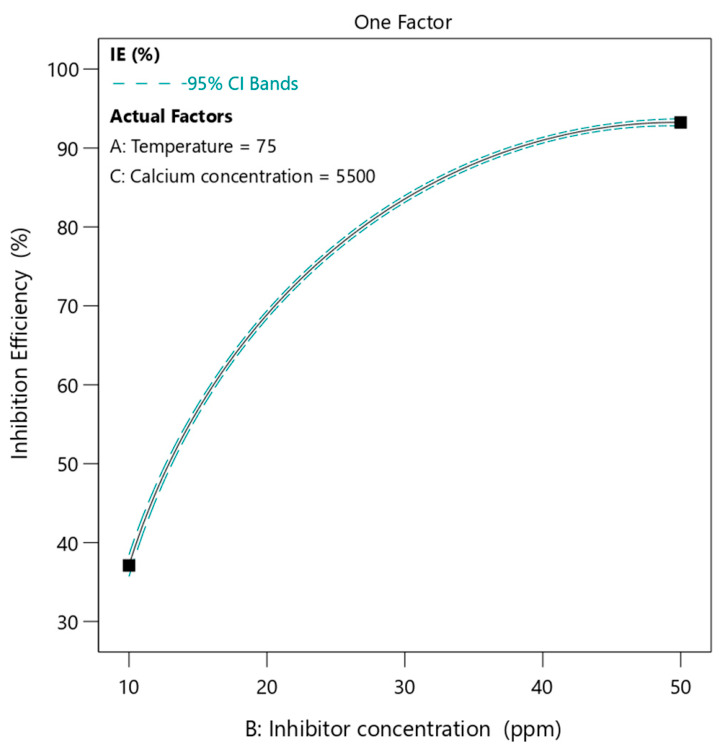
The effect of PPCA concentration on its inhibition efficiency at 75 °C and 5500 ppm of calcium ion content under static condition.

The effect of inhibitor concentration on inhibition efficiency in Figure 7 shows a strong positive and nonlinear trend. As the concentration of phosphino-polycarboxylic acid increases from 10 ppm to 50 ppm, the inhibition efficiency increases significantly from about 37% to approximately 92%. The temperature and calcium ion concentration were kept constant at 75 °C and 5500 ppm, respectively. The relationship is clearly nonlinear. At lower concentrations, the slope is steep, indicating a rapid increase in efficiency with increasing polymer dosage. At higher concentrations, the slope decreases, and the curve approaches a plateau, meaning that further increases in concentration result in smaller improvements in efficiency. This behavior is strongly related to the concept of surface coverage and active site availability [39]. At low inhibitor concentrations, the number of polymer chains in solution is insufficient to effectively interact with all calcium ions or to fully cover the nucleation and growth sites of calcium sulfate crystals. As a result, scale formation proceeds with limited inhibition. As the concentration increases, more polymer chains become available to participate in inhibition mechanisms. These include calcium ion complexation, adsorption onto crystal surfaces, and distortion of crystal growth [65]. The presence of multiple functional groups along the polymer backbone enhances multidentate binding, which significantly improves inhibition performance [32]. From a polymer perspective, the steep initial slope reflects the transition from partial to substantial surface coverage. Once a critical concentration is reached, most of the active crystal growth sites are occupied by polymer molecules. Beyond this point, additional polymer molecules have fewer available sites to interact with, leading to diminishing returns in inhibition efficiency. Furthermore, steric hindrance becomes more effective at higher concentrations. The adsorbed polymer layer creates a physical barrier that prevents further crystal growth and aggregation. However, once this barrier is sufficiently established, additional polymer chains contribute less significantly to performance, explaining the reduced slope at higher concentrations.

#### 3.4.3. Effect of Calcium Ion Concentration

**Figure 8:**
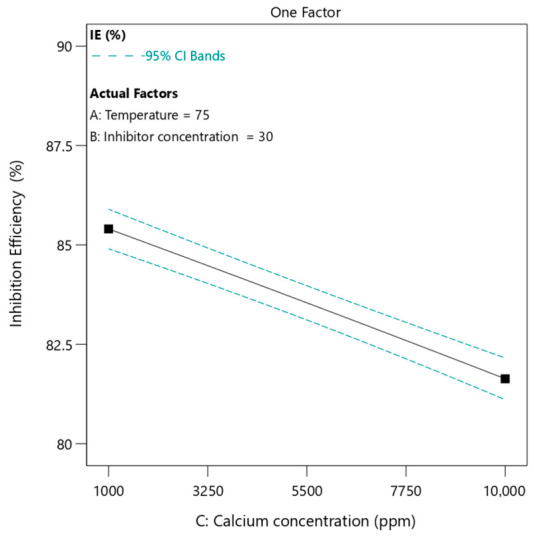
The effect of calcium ion concentration on inhibition efficiency of PPCA at 75 °C and 30 ppm of inhibitor under static condition.

The dependence of inhibition efficiency on calcium ion concentration (Figure 8) shows a decreasing and nearly linear trend. As the calcium ion concentration increases from 1000 ppm to 10,000 ppm, the inhibition efficiency decreases from approximately 85.5% to about 82%. In this case, temperature and inhibitor concentration were maintained con-stant at 75 °C and 30 ppm, respectively. The linear nature of this decrease suggests a direct relationship between calcium ion concentration and scaling tendency. Higher calcium concentrations increase the degree of supersaturation when mixed with sulfate ions. This promotes faster nucleation and growth of calcium sulfate crystals, making inhibition more challenging. From a polymer interaction standpoint, increasing calcium concentration introduces a competitive environment for the functional groups of phosphino-polycarboxylic acid [66,67]. Although the polymer contains multiple binding sites, there is a finite number of active groups available for complexation and adsorption. At higher calcium levels, these sites may become saturated, leaving excess calcium ions free to participate in scale formation. Another important factor is the effect of ionic strength. As calcium concentration increases, the ionic strength of the solution also rises. This leads to compression of the electrical double layer surrounding polymer chains, reducing their effective size and flexibility. Consequently, the polymer’s ability to extend toward and interact with crystal surfaces is diminished. In addition, high calcium concentration may promote intermolecular interactions such as ion bridging between polymer chains. This can reduce the availability of free polymer molecules in solution and decrease their mobility. The combined effect of these phenomena results in a gradual and linear decrease in inhibition efficiency.

The one-factor plots demonstrate that inhibition efficiency is governed by a balance between polymer performance and scale formation driving forces. Temperature and calcium ion concentration promote scale formation, while polymer concentration enhances inhibition efficiency. The results clearly show that phosphino-polycarboxylic acid performs effectively through mechanisms such as ion complexation, surface adsorption, and crystal growth modification. However, its efficiency is sensitive to environmental conditions. The nonlinear effects observed for temperature and inhibitor concentration reflect complex polymer behaviors such as conformational changes, surface saturation, and steric hindrance. In contrast, the linear decrease with calcium concentration indicates a direct competition between scaling ions and inhibitor molecules. These findings highlight the importance of optimizing operating conditions. At moderate temperatures and calcium concentrations, high inhibition efficiency can be achieved with relatively low polymer dosage. However, under more severe conditions, higher inhibitor concentrations are required to compensate for reduced polymer effectiveness and increased scaling tendency.

### 3.5. Combined Effects of Operational Parameters on Inhibition Efficiency

**Figure 9:**
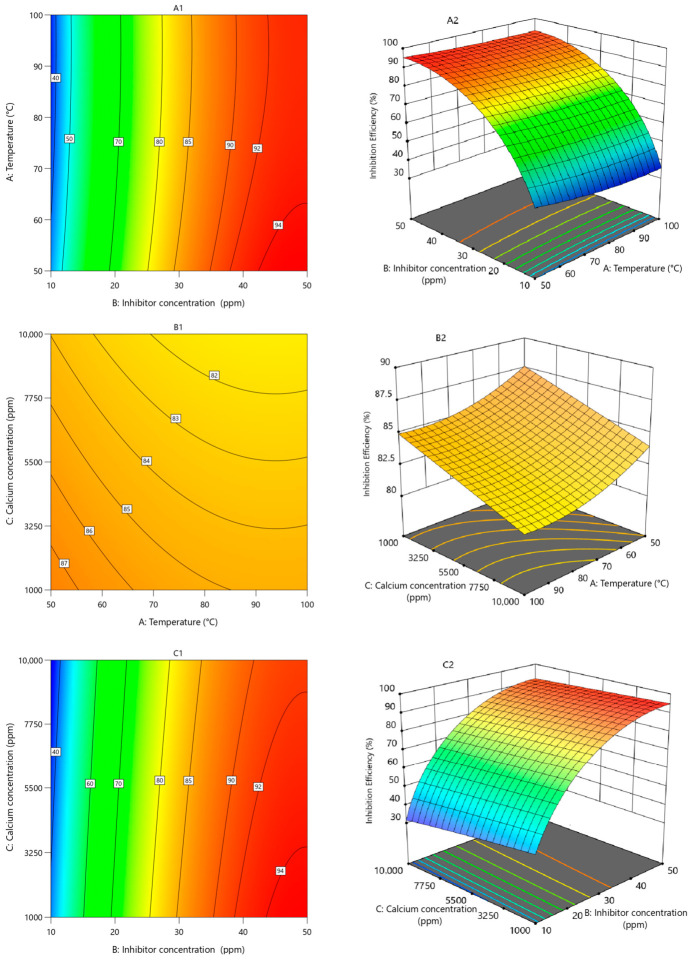
The contour and three-dimensional (3D) response surface plots providing the simultaneous effects of key variables on the inhibition efficiency of calcium sulfate; (**A1**,**A2**) effect of temperature and inhibitor concentration; (**B1**,**B2**) effect of temperature and calcium ion concentration; (**C1**,**C2**) effect of calcium ion concentration and inhibitor concentration.

The contour and three-dimensional (3D) response surface plots are shown in Figure 9. These plots provide a comprehensive understanding of the simultaneous effects of key variables on the inhibition efficiency of calcium sulfate using phosphino-polycarboxylic acid. In all contour and 3D surface plots, the color gradient from blue to red represents increasing inhibition efficiency. Blue/green regions correspond to low efficiency, while yellow, orange, and red regions indicate progressively higher efficiency. The contour lines represent constant inhibition efficiency values. For example, in Figure 9(B1), inhibition efficiency increases from approximately 82% to 87% as the color transitions from yellow to orange/red. Similarly, in Figure 9(C1), blue regions correspond to ~35–50%, green to ~50–75%, yellow/orange to ~75–85%, and red to >85%.

Figure 9(A1,A2) illustrates the combined influence of temperature (50–100 °C) and PPCA concentration (10–50 ppm) on inhibition efficiency, while calcium ion concentration is fixed at 5500 ppm. The results clearly show a strong dependence of inhibition efficiency on inhibitor concentration, along with a secondary influence of temperature. The highest inhibition efficiencies, exceeding 94%, are observed in the region where inhibitor concentration is relatively high, specifically in the range of approximately 43 to 50 ppm, and temperature is relatively low, between 50 and 63 °C. This region is represented by the red-colored zone in the contour plot, indicating optimal performance. Furthermore, it is notable that inhibition efficiencies higher than 90% are maintained across the entire temperature range (50–100 °C) when the inhibitor concentration exceeds approximately 35 ppm. This suggests that at sufficiently high polymer dosage, the effect of temperature becomes less dominant, and the inhibitor is capable of maintaining high performance even under less favorable thermal conditions.

Figure 9(B1,B2) represents the interaction between temperature (50–100 °C) and calcium ion concentration (1000–10,000 ppm), while the inhibitor concentration is fixed at 30 ppm. In this case, the highest inhibition efficiency, approximately 87% and above, is observed in a relatively narrow region corresponding to low temperatures (50–55 °C) and low calcium concentrations (1000 to about 2800 ppm). As temperature increases beyond 55 °C, or calcium concentration increases beyond approximately 2800 ppm, the inhibition efficiency gradually decreases. At high temperatures and high calcium concentrations, the efficiency reaches significantly lower values. This behavior reflects the combined effect of two scale-promoting factors: temperature and calcium ion concentration. Increasing temperature accelerates nucleation and crystal growth rates, while increasing calcium concentration enhances supersaturation. When both parameters increase simultaneously, the scaling tendency becomes very strong, making inhibition more difficult.

Figure 9(C1,C2) illustrates the combined effect of calcium ion concentration (1000–10,000 ppm) and PPCA concentration (10–50 ppm) on inhibition efficiency at a constant temperature of 75 °C. The results show a clear dominance of inhibitor concentration over calcium concentration in determining inhibition performance. At inhibitor dosages greater than approximately 35 ppm, the inhibition efficiency exceeds 90% across the entire range of calcium concentrations (1000–10,000 ppm). This indicates that sufficiently high polymer concentration can effectively counteract the negative impact of high calcium levels. Similarly, inhibition efficiencies greater than 85% are achieved when the inhibitor concentration exceeds approximately 30 ppm, regardless of calcium concentration. This suggests a threshold concentration above which the polymer can provide substantial protection against scale formation. The highest efficiencies, exceeding 94%, are observed at inhibitor concentrations between approximately 44 and 50 ppm, particularly when calcium concentration is in the lower range of 1000 to about 3200 ppm.

### 3.6. Process Optimization

**Figure 10:**
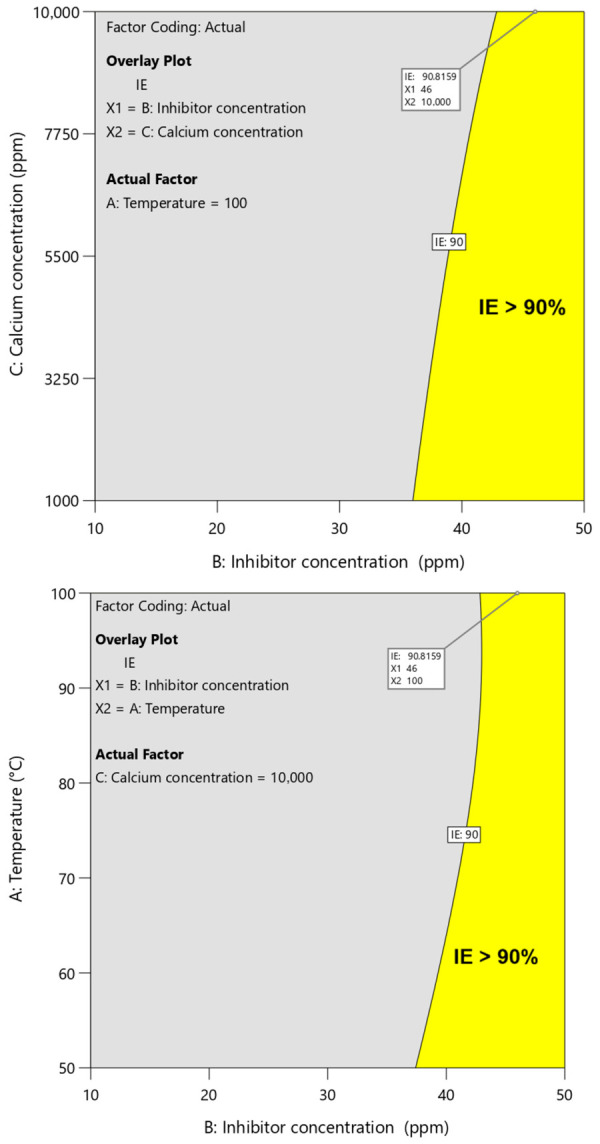
The overlay diagram of the graphical optimization for maximum PPCA scale inhibition efficiency.

**Figure 11:**
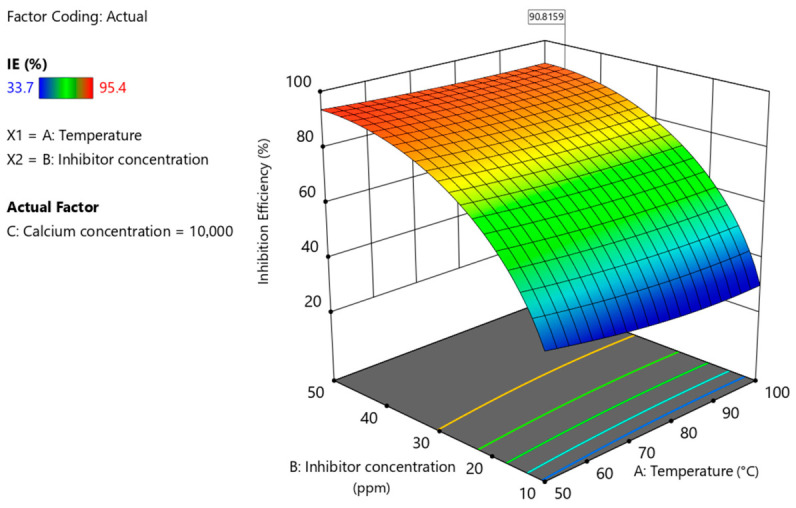
The 3D-plot of the optimization for maximum PPCA scale inhibition efficiency depending on temperature and inhibitor concentration at 10,000 ppm of calcium ion.

The primary objective of this study was to identify the optimal operating conditions that maximize inhibition efficiency. To accomplish this, a numerical optimization procedure was performed targeting an inhibition efficiency greater than 90% (IE > 90%). The outcomes of this optimization are illustrated in Figure 10, which presents the overlay plot obtained from the graphical optimization approach. In this figure, the gray shaded region represents conditions that fail to satisfy the desired efficiency criterion, whereas the highlighted yellow region corresponds to the feasible domain where the optimization target is successfully achieved. Thus, the overlay plot uses discrete color regions to indicate optimization criteria. The gray shaded region represents conditions where inhibition efficiency is below 90%, while the yellow region identifies the optimal operating window where IE% exceeds 90%. This yellow zone defines the range of operational parameters under which the inhibition efficiency exceeds 90%. As observed in Figure 10, even under severe operating conditions (temperature of 100 °C and calcium ion concentration of 10,000 ppm), an inhibitor dosage in the range of 42–50 ppm is sufficient to maintain an inhibition efficiency above 90%. Moreover, the predicted optimal point under these harsh conditions is reported in Table 7 and is indicated by a flag in Figure 10 and Figure 11. Under these optimized conditions, the model predicts an inhibition efficiency of 90.81%, demonstrating the strong performance of PPCA even at elevated temperature and high ionic strength. It should be noted that in Figure 11, The color gradient represents inhibition efficiency ranging from approximately 33.7% (dark blue) to 95.4% (red). Intermediate colors indicate gradual increases in efficiency: blue to green (~33–60%), green to yellow (~60–75%), yellow to orange (~75–85%), and orange to red (>85%).

To further validate the predictive capability of the developed RSM model, an additional experiment was conducted under the optimal operating conditions identified by the model. The comparison between predicted and experimental inhibition efficiency values is presented in Table 8. As shown, the model predicted an inhibition efficiency of 90.81%, while the experimentally obtained value was 91.57%. The deviation between these values is only 0.84%, which is very small and within acceptable experimental error limits. This result provides strong independent validation of the model and confirms its reliability for predicting inhibition efficiency under practical operating conditions. Combined with the excellent agreement observed in the predicted versus actual plot (Figure 5) and the low average prediction error reported in Table 6 (about 1.4%), these findings demonstrate that the developed quadratic model is both accurate and robust.

## 4. Conclusions

This study demonstrated the effectiveness of phosphino-polycarboxylic acid (PPCA) as a polymeric scale inhibitor for calcium sulfate systems under conditions relevant to oilfield applications. By integrating experimental evaluation with Response Surface Methodology (RSM), a reliable predictive model was developed, showing excellent agreement with experimental data (R^2^ = 0.998) and a low prediction error of about 1.4%. The results confirmed that PPCA provides high inhibition efficiency across a wide range of operating conditions, with IE% values reaching up to about 95%. Among the investigated variables, inhibitor concentration was identified as the most influential parameter, with efficiencies exceeding 90% at dosages above approximately 35 ppm. In contrast, increasing temperature (50–100 °C) and calcium ion concentration (1000–10,000 ppm) showed a moderate negative effect on performance. From a practical perspective, the optimization results indicated that inhibition efficiencies above 90% can be maintained over a broad operating window. Notably, even under harsh conditions (100 °C and 10,000 ppm Ca^2+^), PPCA achieved an efficiency of approximately 90.8% at concentrations between 42 and 50 ppm. Overall, the developed RSM model provides a robust and practical tool for predicting and optimizing scale inhibition performance. The findings highlight the strong potential of PPCA for application in oilfield systems and emphasize the importance of selecting appropriate operating conditions to ensure efficient and reliable scale control.

## Figures and Tables

**Table 1:** Ion content of synthetic formation and injection waters.

Ion Type	Ion Concentration (ppm)			
	Formation Water No. 1	Formation Water No. 2	Formation Water No. 3	Injection Water
Sodium	37,258	37,258	37,258	9845
Calcium	1000	6000	10,000	266
Potassium	876	876	876	121
Magnesium	613	613	613	1021
Chloride	82,341	82,341	82,341	19,867
Sulfate	96	96	96	2043
TDS (ppm)	122,184	127,184	131,184	33,163

**Table 2:** The experimental levels of the analyzed parameters.

Parameter	Symbol	Unit	Experimental Levels (Coded and Actual)				
			−1				+1
Temperature	A	°C	50	75			100
Inhibitor concentration	B	ppm	10	20	30	40	50
Calcium concentration	C	ppm	1000	4000 7000			10,000

**Table 3:** The experimental results of PPCA scale inhibition efficiency under static conditions.

Run	IE (%)	Run	IE (%)	Run	IE (%)	Run	IE (%)	Run	IE (%)	Run	IE (%)
1	45.3	11	84.1	21	41.2	31	82.9	41	39.5	51	81.7
2	42.1	12	82.7	22	38.9	32	80.7	42	36.9	52	80.3
3	39.8	13	94.2	23	36.8	33	92.5	43	35.1	53	92.2
4	36.9	14	93.4	24	35	34	91.3	44	33.7	54	91
5	75.8	15	92.8	25	71.3	35	90.1	45	70.8	55	90
6	74.1	16	92	26	68.7	36	88.9	46	68	56	88.7
7	72.5	17	95.4	27	67.1	37	94.8	47	66.7	57	94.5
8	70.7	18	95.1	28	65.8	38	93.9	48	65.2	58	93.4
9	89.4	19	94.6	29	86.2	39	93.1	49	86	59	92.7
10	86.5	20	93.9	30	84.6	40	92.5	50	84.1	60	92.1

**Table 4:** ANOVA results for IE -Model developed by RSM.

Source	Sum of Squares	df	Mean Square	F-Value	*p*-Value	
Model	2.896 × 10^9^	5	5.791 × 10^8^	4674.68	<0.0001	significant
A-T	1.533 × 10^7^	1	1.533 × 10^7^	123.77	<0.0001	
B-Inhibitor concentration	2.621 × 10^9^	1	2.621 × 10^9^	21,153.19	<0.0001	
C-Calcium concentration	2.346 × 10^7^	1	2.346 × 10^7^	189.33	<0.0001	
A^2^	2.248 × 10^6^	1	2.248 × 10^6^	18.15	<0.0001	
B^2^	2.340 × 10^8^	1	2.340 × 10^8^	1888.95	<0.0001	
Residual	6.690 × 10^6^	54	1.239 × 10^5^			
Cor Total	2.902 × 10^9^	59				

**Table 5:** Fit statistics for IE-model.

№	Fit Statistics		IE-Model
1	Coefficient of determination	R^2^	0.9977
2	Adjusted coefficient of determination	Adj-R^2^	0.9975
3	Predicted coefficient of determination	Pred-R^2^	0.9971
4	Adequate precision	Ad.P	194.1393
5	Coefficient of variation	C.V. %	2.37

**Table 6:** The comparison of actual and predicted values of inhibition efficiency of PPCA.

Run Order	Actual Value	Predicted Value	PE% $(\left|\frac{actual-predicted}{actual}\right|)\times 100\%$
1	45.3	46.8	3.2%
2	42.1	44.1	4.9%
3	39.8	41.3	3.9%
4	36.9	38.3	3.8%
5	75.8	73.9	2.4%
6	74.1	72.5	2.2%
7	72.5	71.0	2.1%
8	70.7	69.4	1.8%
9	89.4	87.6	2.0%
10	86.5	86.4	0.1%
11	84.1	85.2	1.3%
12	82.7	84.0	1.5%
13	94.2	94.7	0.5%
14	93.4	93.6	0.2%
15	92.8	92.5	0.3%
16	92	91.4	0.7%
17	95.4	96.8	1.5%
18	95.1	95.8	0.7%
19	94.6	94.7	0.1%
20	93.9	93.6	0.3%
21	41.2	41.8	1.5%
22	38.9	38.8	0.3%
23	36.8	35.5	3.6%
24	35	31.7	9.4%
25	71.3	71.2	0.1%
26	68.7	69.7	1.4%
27	67.1	68.1	1.5%
28	65.8	66.5	1.0%
29	86.2	85.4	0.9%
30	84.6	84.2	0.5%
31	82.9	82.9	0.0%
32	80.7	81.6	1.2%
33	92.5	92.7	0.2%
34	91.3	91.6	0.3%
35	90.1	90.4	0.4%
36	88.9	89.3	0.4%
37	94.8	94.9	0.1%
38	93.9	93.8	0.1%
39	93.1	92.7	0.4%
40	92.5	91.6	1.0%
41	39.5	40.7	3.1%
42	36.9	37.6	1.9%
43	35.1	34.1	2.8%
44	33.7	30.2	10.5%
45	70.8	70.6	0.2%
46	68	69.1	1.6%
47	66.7	67.5	1.2%
48	65.2	65.8	1.0%
49	86	84.9	1.2%
50	84.1	83.7	0.5%
51	81.7	82.4	0.9%
52	80.3	81.2	1.1%
53	92.2	92.3	0.1%
54	91	91.1	0.1%
55	90	90.0	0.0%
56	88.7	88.8	0.2%
57	94.5	94.5	0.0%
58	93.4	93.4	0.0%
59	92.7	92.3	0.4%
60	92.1	91.2	1.0%
			Average = 1.4%

**Table 7:** Optimal values of temperature, PPCA concentration, and calcium ion concentration for maximum inhibition performance under harsh conditions.

Parameter	Variable	Unit	Optimal Value
Temperature	A	°C	100
Inhibitor concentration	B	ppm	46
Calcium concentration	C	ppm	10,000

**Table 8:** Experimental validation of the RSM model by comparing predicted and measured inhibition efficiency under optimal operating conditions.

Parameter	Unit	Value
Temperature	°C	100
Inhibitor concentration	ppm	46
Calcium concentration	ppm	10,000
Inhibition efficiency (predicted by model)	%	90.81
Inhibition efficiency (experimental)	%	91.57
Deviation between predicted and experimental values	%	0.84

## Data Availability

The data used is provided in the article and can be used.

## Appendix A

**Table A1:** RSM experimental design of static jar test for PPCA inhibition efficiency evaluation.

Run	Factor		
	A: Temperature	B: Inhibitor Concentration	C: Calcium Concentration
	°C	ppm	ppm
1	50	10	1000
2	50	10	4000
3	50	10	7000
4	50	10	10,000
5	50	20	1000
6	50	20	4000
7	50	20	7000
8	50	20	10,000
9	50	30	1000
10	50	30	4000
11	50	30	7000
12	50	30	10,000
13	50	40	1000
14	50	40	4000
15	50	40	7000
16	50	40	10,000
17	50	50	1000
18	50	50	4000
19	50	50	7000
20	50	50	10,000
21	75	10	1000
22	75	10	4000
23	75	10	7000
24	75	10	10,000
25	75	20	1000
26	75	20	4000
27	75	20	7000
28	75	20	10,000
29	75	30	1000
30	75	30	4000
31	75	30	7000
32	75	30	10,000
33	75	40	1000
34	75	40	4000
35	75	40	7000
36	75	40	10,000
37	75	50	1000
38	75	50	4000
39	75	50	7000
40	75	50	10,000
41	100	10	1000
42	100	10	4000
43	100	10	7000
44	100	10	10,000
45	100	20	1000
46	100	20	4000
47	100	20	7000
48	100	20	10,000
49	100	30	1000
50	100	30	4000
51	100	30	7000
52	100	30	10,000
53	100	40	1000
54	100	40	4000
55	100	40	7000
56	100	40	10,000
57	100	50	1000
58	100	50	4000
59	100	50	7000
60	100	50	10,000
